# Galangin, a novel dietary flavonoid, attenuates metastatic feature via PKC/ERK signaling pathway in TPA-treated liver cancer HepG2 cells

**DOI:** 10.1186/s12935-015-0168-2

**Published:** 2015-02-04

**Authors:** Shang-Tao Chien, Ming-Der Shi, Yi-Chieh Lee, Chou-Chia Te, Yuan-Wei Shih

**Affiliations:** Department of Pathology, Kaohsiung Armed Forces General Hospital, Kaohsiung, 80284 Taiwan; Department of Medical Laboratory Sciences and Biotechnology, Fooyin University, Kaohsiung, 83102 Taiwan; Department of Medical Technology, Kaohsiung Veterans General Hospital Tainan Branch, Tainan, 71051 Taiwan; Department of Medical Laboratory Science and Biotechnology and Graduate Institute of Biological Technology, Chung Hwa University of Medical Technology, Tainan, 71703 Taiwan; Department of Nursing, Chung Hwa University of Medical Technology, Tainan, 71703 Taiwan; Department of Biological Science and Technology and Graduate Institute of Biomedical Science, Chung Hwa University of Medical Technology, Tainan, 71703 Taiwan; Department of Food Nutrition, Chung Hwa University of Medical Technology, Tainan, 71703 Taiwan

**Keywords:** Galangin, TPA, Invasion, Migration, PKC-α, ERK, MMP-2, MMP-9

## Abstract

**Background:**

Galangin (3,5,7-trihydroxyflavone) is a flavonoid compound found in high concentration in lesser galangal. The objective of this study was to investigate the ability of galangin to inhibit 12-O-tetradecanoylphorbol-13-acetate (TPA)-induced the invasion and metastasis of HepG2 liver cancer cells.

**Results:**

First, using a cell-matrix adhesion assay, immunofluorescence assay, transwell-chamber invasion/migration assay, and wound healing assay, we observed that galangin exerted an inhibitory effect on TPA-induced cell adhesion, morphology/actin cytoskeleton arrangement, invasion and migration. Furthermore, the results of gelatin zymography and reverse transcriptase polymerase chain reaction (RT-PCR) assays showed that galangin reduced the TPA-induced enzyme activity of matrix metalloproteinase-2 (MMP-2) and matrix metalloproteinase-9 (MMP-9) in HepG2 cells; moreover, the messenger RNA level was downregulated. We also observed through a Western blotting assay that galangin strongly inhibited the TPA-induced protein expressions of protein kinase Cα (PKCα), protein kinase Cδ (PKCδ), phosphorylated extracellular signal-regulated kinase 1/2 (ERK1/2), the phospho-inhibitor of kappaBα (phospho-IκBα), c-Fos, c-Jun, and nuclear factor kappa B (NF-κB). Next, galangin dose-dependently inhibited the binding ability of NF-κB and activator protein 1 (AP-1) to MMP-2/MMP-9 promoters, respectively, resulting in the suppression of MMP-2/MMP-9 enzyme activity.

**Conclusions:**

The results revealed that galangin effectively inhibited the TPA-induced invasion and migration of HepG2 cells through a protein kinase C/extracellular signal-regulated kinase (PKC/ERK) pathway. Thus, galangin may have widespread applications in clinical therapy as an anti-metastatic medicament.

## Introduction

Malignant neoplasm, generally known as cancer, is currently considered one of the most deadly types of diseases. The leading cause of death in patients with cancer is tumour metastasis. Hepatocellular carcinoma (HCC) is amongst the most common types of malignant tumours in the tropics and East Asian countries, including Taiwan. Surgical resection remains the treatment of choice, but because of the high rate of metastasis and poor prognoses, the number of deaths is almost the same as the number of new cases occurring each year worldwide [1]. Metastasis of HCC to distinct sites or organs, such as the lymph nodes, lungs, spleen, peritoneum, and bones, is a major cause of death [2].

Tumour metastasis is a complex process during which cancer cells undergo a series of alterations at intracellular and extracellular levels, including changes that (1) damage intercellular interaction, (2) increase cancer cells and extracellular matrix (ECM) interaction, (3) damage ECM components, and (4) increase the invasion and migration of cancer cells. In addition, metastasis involves the over-expression of proteolytic enzymes, such as matrix metalloproteinases (MMPs) [3]. MMPs are enzymes involved in ECM degradation in physiological processes, such as tissue remodelling and embryonic development. Studies have shown that MMP-2 and MMP-9 are particularly crucial in tumour metastasis, the process of which requires the decomposition of the ECM [4,5]. Furthermore, TPA is a diester of phorbol and a potent tumour promoter often employed in biomedical research to activate the signal transduction enzyme PKC [6]. PKC isoforms comprise a family of serine/threonine kinases that can be activated as major signalling transduction enzymes to respond to extracellular signals, such as those by diacylglycerol and calcium ions, that are generated by ligand-receptor interactions with the nucleus at the cell surface [7]. In addition, PKC isoforms are involved in signalling transduction pathways for numerous biological processes, such as proliferation, survival, angiogenesis, metastasis, and tumourigenesis [8-10]. Previous studies has demonstrated that TPA controls the expressions of MMPs by modulating the activation of transcription factors, such as NF-kB and AP-1, through phosphatidylinositide-3 kinase/Akt (PI3K/Akt), c-Jun N-terminal kinase (JNK), p38, and ERK signalling pathways [11]. PI3K activation leads to the phosphorylation of phosphatidyl-inositides, which activates the primary downstream target Akt that apparently plays various crucial roles in regulating cellular growth, differentiation, adhesion, inflammatory reactions, and invasion [12,13]. Three major mammalian mitogen-activated protein kinases (MAPKs) are ERK, JNK, and p38. Various MAPKs are activated in response to different extracellular stimuli and have distinct downstream targets, thus serving unique roles in cellular responses. The activation of MAPKs has been shown in other systems to be the mechanism crucial for promoting the production of MMPs, which are vital for cell proliferation, invasion, and neovascularization [14-16]. In addition, the gene expressions of MMPs are primarily regulated by transcriptional factors, such as NF-κB and AP-1 (a heterodimer consisting of protein molecules from the Fos and Jun protein families).

Galangin is a naturally occurring flavonoid found in *Alpinia officinarum* (lesser galangal). Flavonoids are well known antioxidants, which can protect cells from being damaged by free radicals [17-19], and are believed to exert inhibitory effects on cancer cells [20,21]. Previous studies have demonstrated that galangin exhibits anti-proliferative and apoptotic effects on the growth of cancer cells that originate from human leukemia cells [22,23]. Although galangin may inhibit the growth of various cancers by inducing antiproliferation and apoptosis in cancer cells, the precise effect and related molecular mechanism of galangin involved in the TPA-induced metastatuc feature of HepG2 liver cancer cells remains unclear.

## Material and methods

### Reagents and antibodies

Galangin (purity ≥ 99%) was purchased from Extrasynthese (Genay, France); dimethylsulfoxide (DMSO), Tris–HCl, ethylenediaminetetraacetic acid (EDTA), sodium dodecyl sulphate (SDS), phenylmethylsulfonyl fluoride, bovine serum albumin (BSA), gelatin, leupeptin, Nonidet P-40, deoxycholic acid and sodium orthovanadate were purchased from Sigma-Aldrich Chemical Co. (St. Louis, MO, USA); A protein assay kit was obtained from Bio-Rad Labs. (Hercules, CA, USA). Dulbecco’s phosphate buffer solution (PBS), fetal bovine serum (FBS), trypsin-EDTA, and powdered Dulbecco’s modified Eagle’s medium (DMEM) were purchased from Gibco-BRL (Gaithersburg, MD, USA). Matrigel was obtained from BD Transduction Laboratories (San Diego, CA, USA). Antibodies against Akt, ERK1/2, JNK/SAPK, and p38 MAPK, proteins, and phosphorylated proteins were purchased from Cell Signalling Technology (Beverly, MA, USA). An enhanced chemiluminescence (ECL) kit was purchased from Amersham Life Science (Amersham, UK).

### Cell culture and galangin treatment

Human nonmalignant Chang liver cells, human hepatocellular carcinoma HepG2 cells, and human hepatocellular carcinoma Hep3B cells were maintained in DMEM medium. Human gastric adenocarcinoma AGS cells was maintained in RPMI-1640 medium. Above-mentioned cell lines were obtained from BCRC (Bioresource Collection and Research Center in Hsin-Chu, Taiwan). All cells were cultured at 37°C in a humidified atmosphere of 5% CO_2_-95% air. In a medium supplemented with 10% FBS and antibiotics (100 U/ml of penicillin and 100 mg/ml of streptomycin). Adherent cells were detached through incubation with trypsin. For galangin treatment, the stock solution of galangin was dissolved in DMSO and sterilised through filtration by using 0.2-μm disc filters. Appropriate amounts of the stock solution (10 mg/ml in DMSO) of galangin were added to the cultured medium to achieve the indicated concentrations.

### Cell viability (MTT assay)

To measure the effect of galangin on cell viability, the Chang liver, AGS, Hep3B, and HepG2 cells were seeded in 24-well plates (1 × 10^5^ cells/well) for 16–18 h. The cells were treated with or without various concentrations of galangin (0, 1, 2.5, 5, 10, 15, 20, 25, and 30 μM) for 24 h and 48 h. Treatment at each concentration was repeated three times. To further investigate whether galangin and/or TPA influence cell viability, HepG2 cells were treated with the presence or absence of drugs (70 nM TPA and 5 μM galangin) for 24 h. After the exposure period, the medium was removed and the cells were washed with PBS. The medium was changed, incubated with an MTT solution (5 mg/ml/well) for 4 h, and then removed. Formazan was solubilised in isopropanol and measured spectrophotometrically at 563 nm. The percentage of viable cells was estimated by comparing the number of viable treated cells with the number of viable untreated control cells.

### Cell-matrix adhesion assay

HepG2 cells were pretreated with 70 nM TPA and incubated in various concentrations of galangin (0, 1, 2.5, and 5 μM) for 24 h. The cells were seeded at a density of 1 × 10^5^ cells/ml in a 24-well plate, coated with 500 μl of type IV collagen (10 μg/ml), and cultured for 30 min. Nonadherent cells were removed by using PBS washes, and adherent cells were fixed in ethanol. After being stained with 0.1% crystal violet, the fixed cells were lysed in 0.2% Triton X-100, and measured spectrophotometrically at 550 nm.

### Immunofluorescence assay

To determine the effect of galangin on cell morphology and actin stress fibers, HepG2 cells (4 × 10^5^ cells/well) were plated in 6-well plates, grown for 16–18 h, stimulated with 70 nM TPA for 12 h, and incubated in various concentrations of galangin (0, 1, 2.5, and 5 μM) for 24 h. After the exposure period, the medium was removed, and the cells were washed with Ca^2+^/Mg^2+^ free PBS. The cells were then fixed with 4% paraformaldehyde in Ca^2+^/Mg^2+^_−_free PBS for 15 min and incubated with 0.5% Triton X-100 in Ca^2+^/Mg^2+^ free PBS for 5 min. The cells were incubated with 1% BSA and 0.5% Triton X-100 in Ca^2+^/Mg^2+^_−_free PBS for 1 h (blocking) and then incubated with 200 U/ml Alexa flour 488-phallodin for 1 h to stain the actin filaments. Fluorescent images were captured using a BX51 fluorescence microscope (Olympus, Tokyo, Japan).

### Transwell-chamber invasion and migration assay

The invasion assay was performed by using Hanging Cell Culture-inserts (BD Biosciences; pore size, 8 μm) in a 6-well plate. The ability of HepG2 cells to pass through filters coated with Matrigel was measured using a transwell-chamber invasion assay. Matrigel was diluted to 200 μg/ml with distilled cold-filtered water and applied to the upper surface of the filter inserts. Briefly, HepG2 cells were stimulated with 70 nM TPA for 12 h and incubated in various concentrations of galangin (0, 1, 2.5, and 5 μM) for 24 h. After 24 h, the cells were detached using trypsin and resuspended in a serum-free medium. A medium containing 10% FBS medium was applied to the lower chamber as a chemoattractant, and cells were seeded on the upper filter at a density of 1 × 10^5^ cells/ml in the serum-free medium. The 6-well plate was incubated for 24 h at 37°C in 5% CO_2_, the filter inserts were removed from the wells, and the cells on the upper surface of the filter were wiped with a cotton-tipped swab. Filters were fixed with methanol for 10 min and stained with Giemsa dye for 1 h. The cells that invaded the lower surface of the filter were counted under a light microscope. The data were expressed as the average number of cells attached to the bottom surface from randomly chosen fields. Each experiment was conducted in triplicate.

To measure the migrative ability of HepG2 cells, cells were seeded on the upper surface of the filter inserts with 8-μm pore polycarbonate filters that were not coated with Matrigel. The migrative cells were treated and measured as described in the invasion assay.

### Wound healing assay

To determine cell motility, HepG2 cells (1 × 10^5^ cells/ml) were seeded in a 6-well tissue culture plate by using DMEM medium containing 10% FBS and grown to 80-90% confluence. After the medium was aspirated, the center of the cell monolayer was scraped using a sterile micropipette tip to create a denuded zone (gap) featuring an even width. Subsequently, cellular debris was washed with PBS, and the HepG2 cells were pretreated with 70 nM TPA for 12 h and incubated in various concentrations of galangin (0, 1, 2.5, and 5 μM) for 24 h. Wound closure was monitored and photographed using an Olympus CKX-41 inverted microscope and an Olympus E-410 camera. To quantify the migrated cells, pictures of the initial wounded monolayers were compared with the corresponding pictures of cells at the end of the incubation period. Artificial lines that corresponded to the cutting edges were drawn on pictures of the original wounds and overlain on the pictures of the cultures after incubation. Cells that migrated across white lines were counted in 6 random fields from each triplicate treatment; data were presented as the mean ± standard deviation (SD).

### Gelatin zymography assay

The activities of MMP-2 and MMP-9 were assayed by gelatin zymography. HepG2 cells (4 × 10^5^ cells/well) were plated in 6-well plates, stimulated with 70 nM TPA for 12 h, and incubated in various concentrations of galangin (0, 1, 2.5, and 5 μM) for 24 h. The conditioned medium was collected and gelatin zymography was performed to examine the activities of MMP-2 and MMP-9. Samples were mixed with a loading buffer and electrophoresed on an 8% SDS-polyacrylamide gel containing 0.1% gelatin. Electrophoresis was performed at 140 and 110 V for 3 h. Gels were washed twice with a zymography washing buffer (2.5% Triton X-100 in double-distilled H_2_O) at room temperature to remove SDS. The cells were incubated at 37°C for 12–16 h in zymography reaction buffer (40 mM Tris–HCl (pH 8.0), 10 mM CaCl_2_, 0.02% NaN_3_), stained with Coomassie blue R-250 (0.125% Coomassie blue R-250, 0.1% amino black, 50% methanol, 10% acetic acid) for 1 h, and destained with a destaining solution (20% methanol, 10% acetic acid, 70% double-distilled H_2_O). Nonstaining bands representing the levels of the latent forms of MMP-2 and MMP-9 were quantified through densitometer measurement by using a digital imaging analysis system.

### Isolation of total RNA, reverse transcriptase polymerase chain reaction (RT-PCR), and DNA electrophoresis

Total RNA was isolated from HepG2 cells by using the total RNA Extraction Midiprep System (Viogene Bio-Tek Corporation, Taiwan). Total RNA (2 μg) was transcribed to 20 μl cDNA with 1 μl dNTPs (2.5 mM), 1 μl oligo dT (10 pmole/μl), and 1 μl RTase (200 U), 1 μl RNase inhibitor and 5× reaction buffer. The appropriate primers (sense of MMP-2, 5′-GGCCCTGTCACTCCTGAGAT-3′, nt 1337–1356; antisense of MMP-2, 5′-GGCATCCAGGTTATCGGGGA-3′, nt 2026–2007; sense of MMP-9, 5′-AGGCCTCTACAGAGTCTTTG-3′, nt 1201–1220; antisense of MMP-9, 5′-CAGTCCAACAAGAAAGGACG-3′, nt 1700–1683; sense of GADPH, 5′-CGGAGTCAACGGATTGGTGTT-3′, nt 94–126; antisense of 5′-AGCCTTCTCCATGGTTGGTGAAGAC-3′, nt 399–375) were used for PCR amplifications. The PCR was performed by using Platinum Taq polymerase (Invitrogen) under the following conditions: 30 cycles at 94°C for 1 min, 59°C (MMP-2) or 60°C (MMP-9 and GAPDH) for 1 min, 72°C for 1 min, and 72°C for 10 min.

### Western blotting assay

The membrane as well as cytosolic and nuclear fractions of cells were prepared as described previously [24]. Western blotting was performed as follows. Denatured samples (50 μg extracted protein) were resolved on 10-12% SDS-PAGE gels. The proteins were transferred onto nitrocellulose membranes. Nonspecific binding of the membranes was blocked with Tris-buffered saline (TBS) containing 1% (w/v) nonfat dry milk and 0.1% (v/v) Tween-20 (TBST) for longer than 2 h. Membranes were washed 3 times with TBST for 10 min and incubated with an appropriate dilution of specific primary antibodies in TBST overnight at 4°C. The membranes were then washed with TBST and incubated with secondary antibody (i.e., horseradish peroxidase-conjugated goat antimouse or antirabbit immunoglobulin G) for 1 h. After the membranes were again washed three times in TBST for 10 min, the bands were detected by performing ECL by using ECL Western blotting detection reagents and exposed ECL hyperfilm in a FUJFILM Las-3000 mini image-analysis system (Tokyo, Japan). Proteins were quantitatively determined through densitometry by using FUJFILM-Multi Gauge V3.0 software.

### Electrophoretic mobility shift assay

Cell nuclear proteins were extracted using a nuclear extract buffer and measured using an electrophoretic mobility shift assay (EMSA). Cells (1 × 10^5^/ml) were collected in a PBS buffer (pH 7.4) and centrifuged at 2000 g for 5 min at 4°C. The cells were lysed with buffer A (10 mM HEPES, 1.5 mM MgCl_2_, 10 mM KCl, 0.5 mM DTT, and 0.5 mM PMSF〔pH 7.9〕 containing 5% NP-40) for 10 min on ice and were subsequently subjected to vortexing to shear the cytoplasmic membranes. The lysates were centrifuged at 2000 g for 10 min at 4°C. The pellet containing the nuclei was extracted using high salt buffer B (20 mM HEPES, 420 mM NaCl, 1.5 mM MgCl_2_, 0.5 mM DTT, 0.5 mM PMSF, 0.2 mM EDTA, and 25% glycerol) for 15 min on ice. The lysates were centrifuged at 13000 g for 10 min at 4°C. The supernatant containing the nuclear proteins was collected and frozen at −80°C until use. The protein content of nuclear fractions was determined using the Bio-Rad protein assay. A 5 μg aliquot of nuclear proteins was mixed with biotin-labeled NF-κB or AP-1 oligonucleotide probes for 15 min at room temperature or with oligonucleotides (sense of NF-κB, 5′-AGTTGAGGGGACTTTCCCAGGC-3′, antisense of NF-κB, 3′-TCAACTCCCCTGAAAGGGTCCG-5′; sense of AP-1, 5′-CGCTTGATGACTCAGCCGGAA-3′, antisense of AP-1, 3′-GCGAACTACTGAGTCGGCCTT-5′). DNA probes were added to 10 μl binding reactions containing double-distilled H_2_O, 5 μg nuclear proteins, 1 μl poly (dI-dC), 1 μl biotin-labeled double stranded NF-κB or AP-1 oliginucleotides, and 2 μl of 10-fold binding buffer into a microcentrifuge tube and incubated for 15 min at room temperature. Specific competition binding assays were performed by adding 200-fold excess of unlabeled probe as a specific competitor. After protein-DNA complexes formation, samples were loaded onto a 6% nondenaturing polyacrylamide gel in a 0.5% TBE buffer and transferred to positively charged nitrocellulose membranes (Milipore, Bedford, MA) by usng a transfer blotting apparatus, and crosslinked in a Stratagene crosslinker. Gel shifts were visualised by first using streptavidin-horseradish peroxidase and then chemiluminescent detection.

### Statistical analysis

Data were expressed as the means ± SD of three independent experiments and analysed using the Student’s *t*-test (Sigmaplot 2001, SPSS Inc., Chicago, IL, USA). Significant differences were established at *P* ≤ 0.05.

## Results

### Galangin inhibits the viability of HepG2, Hep3B, and AGS cells

**Figure 1:**
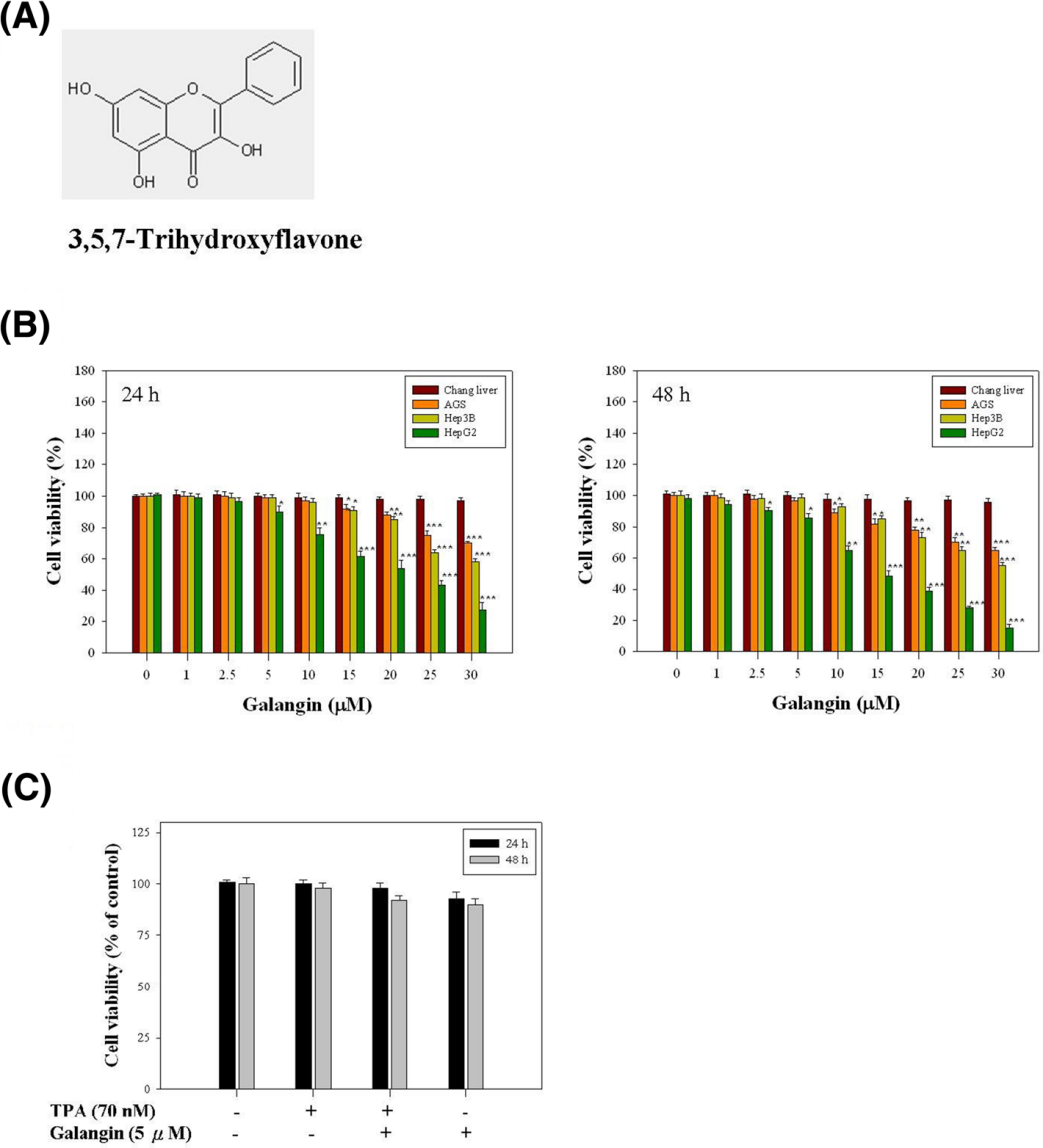
**Effect of galangin on the viability in four cell lines, Chang liver, AGS, Hep3B, and HepG2 cells. (A)** Chemical structure of galangin. **(B)** Four cell lines were treated with or without various concentrations of galangin (0, 1, 2.5, 5, 10, 15, 20, 25, and 30 μM) for 24 and 48 h, separately. **(C)** HepG2 cells were treated with or without of drugs (70 nM TPA and 5 μM galangin) for 24 and 48 h. Thereafter, cell viability was determined by MTT assay. The survival cell number was directly proportional to formazan, which was measured spectrophotometrically at 563 nm. Values represent mean ± SD of three independent experiments. *p < 0.05, **p < 0.01, ***p < 0.001 compared with the untreated control (dose 0).

Figure 1A lists the chemical structure of galangin. In this study, we first examined the effect of galangin on cell viability in four cell lines, Chang liver, AGS, Hep3B, and HepG2 cells. As shown in Figure 1B, galangin exhibited a dose- and time-dependent inhibitory effect on the cell viability of four cancer cell lines. The strongest potency of galangin on the viability inhibitory effect of cancerous cells was toward HepG2 cells. Compared to the control group, after 24 and 48 h of treatment with galangin at concentrations between 0 and 5 μM, the cell viability was not significantly altered, indicating galangin was not toxic to HepG2 cells at these dosages. When cells were treated with 10–30 μM galangin for 24 and 48 h, cell viability was significantly reduced. These results demonstrated that treatment with galangin at doses exceeding 5 μM for 24 and 48 h resulted in dose- and time- dependent loss of cell viability in HepG2 cells; however, treatment with doses lower than 5 μM for 24 and 48 h did not cause cytotoxicity. Moreover, galangin did not significantly inhibit the viability of Chang liver cells at the same concentrations. Next, we used the HepG2 cell line to perform subsequent experiments, confirming that treating HepG2 cells first with 70 nM TPA and then with 5 μM galangin did not alter the viability and thereby ensuring that the investigation of cell invasion and migration (described as follows) was reliable (Figure 1C).

### Galangin inhibited TPA-induced cell adhesion, morphology/actin cytoskeleton arrangement, invasion, and migration in HepG2 cells

**Figure 2:**
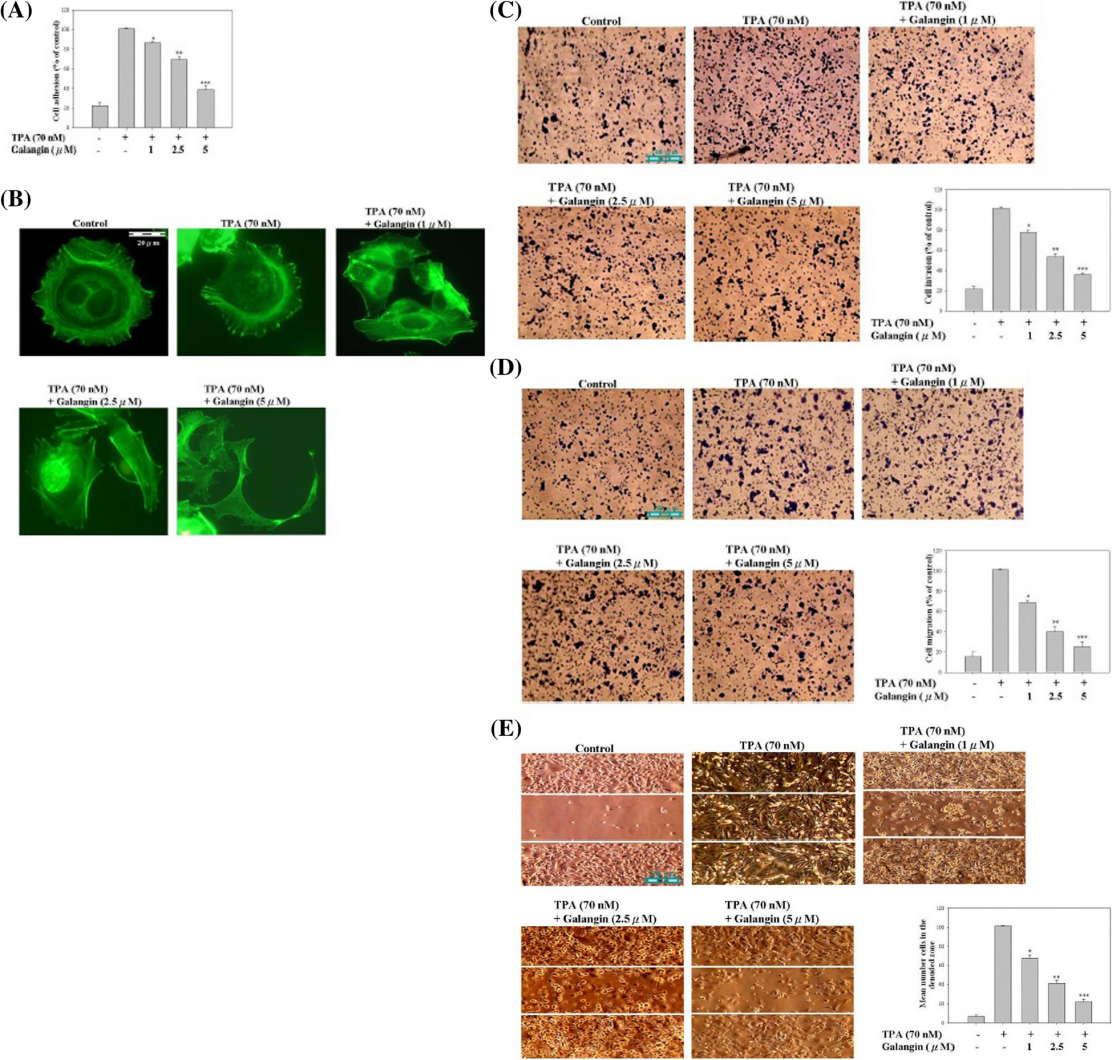
**Effect of galangin on TPA-induced cell-matrix adhesion, cell morphology/actin cytoskeleton arrangement, invasion, and migration in HepG2 cells.** Cells were treated with 70 nM TPA for 12 h and incubated in various concentrations of galangin (0, 1, 2.5, and 5 μM) for 24 h, and then were analysed for **(A)** cell-matrix adhesion, **(B)** immunofluorescence, scale bar: 20 μm, **(C)** transwell-chamber invasion, scale bar: 100 μm, **(D)** transwell-chamber migration, scale bar: 100 μm, and **(E)** wound-healing, scale bar: 100 μm. The aforementioned methods were described in “Materials and methods” section. Values are expressed as mean ± SD of three independent experiments. *p < 0.05, **p < 0.01, ***p < 0.001 compared with the untreated control (dose 0).

Cell adhesion, the binding of one cell to another cell or to the ECM, is a critical process through which cancer cells establish new tumours in the body. Therefore, we used a cell-matrix adhesion assay to investigate the ability of galangin to inhibit cancer cell adhesion. After treating cells with TPA for 12 h and with galangin in various concentrations (1, 2.5, and 5 μM) for 24 h, we observed that galangin inhibited the adhesion of TPA-treated HepG2 cells in a dose-dependent manner (Figure 2A); particularly, HepG2 cells were treated with 5 μM galangin for 24 h, exhibited substantial decreases in cell adhesion ability.

To regulate migratory and invasive behaviour, tumour cells must deform themselves, a process that requires the activity of the cytoskeleton. Therefore, cytoskeleton functions play vital roles in tumour cell growth, invasion of surrounding tissue, and metastasis to new sites. We used the immunofluorescence assay to investigate the influence of galangin on the morphology of cancer cells, first treating HepG2 cells with TPA for 12 h and then with galangin in various concentrations (1, 2.5, and 5 μM) for 24 h. A comparison of these cells to control cells treated only with TPA showed that the cytoskeletons of the HepG2 cells became elongated and contracted as the concentration of galangin increased (Figure 2B).

Tumour invasion and migration are the primary causes of morbidity in patients with cancer. To clarify the influence of galangin on the invasion ability and motility of cancer cells, we used a transwell chamber invasion and migration assay, observing that galangin (5 μM) significantly inhibited TPA-induced invasion and migration by 64.3 % and 75%, respectively, compared with TPA treatment alone (Figure 2C and D). Further examination the effect of galangin on HepG2 cell migration was determined using a wound-healing assay. As shown in Figure 2E, the TPA-induced cell motility of HepG2 cells was significantly increased compared to the untreated cells. Treatment with 1 or 2.5 μM galangin reduced the TPA-induced motility of cells, and 5 μM galangin significantly blocked cell motility. These results demonstrated that galangin may be used to suppress tumour adhesion, F-actin patterns, and the invasion and migration of highly metastatic HepG2 cells at various concentrations.

### Galangin inhibited TPA-induced MMP-2, MMP-9 enzyme activity and mRNA expression in HepG2 cells

**Figure 3:**
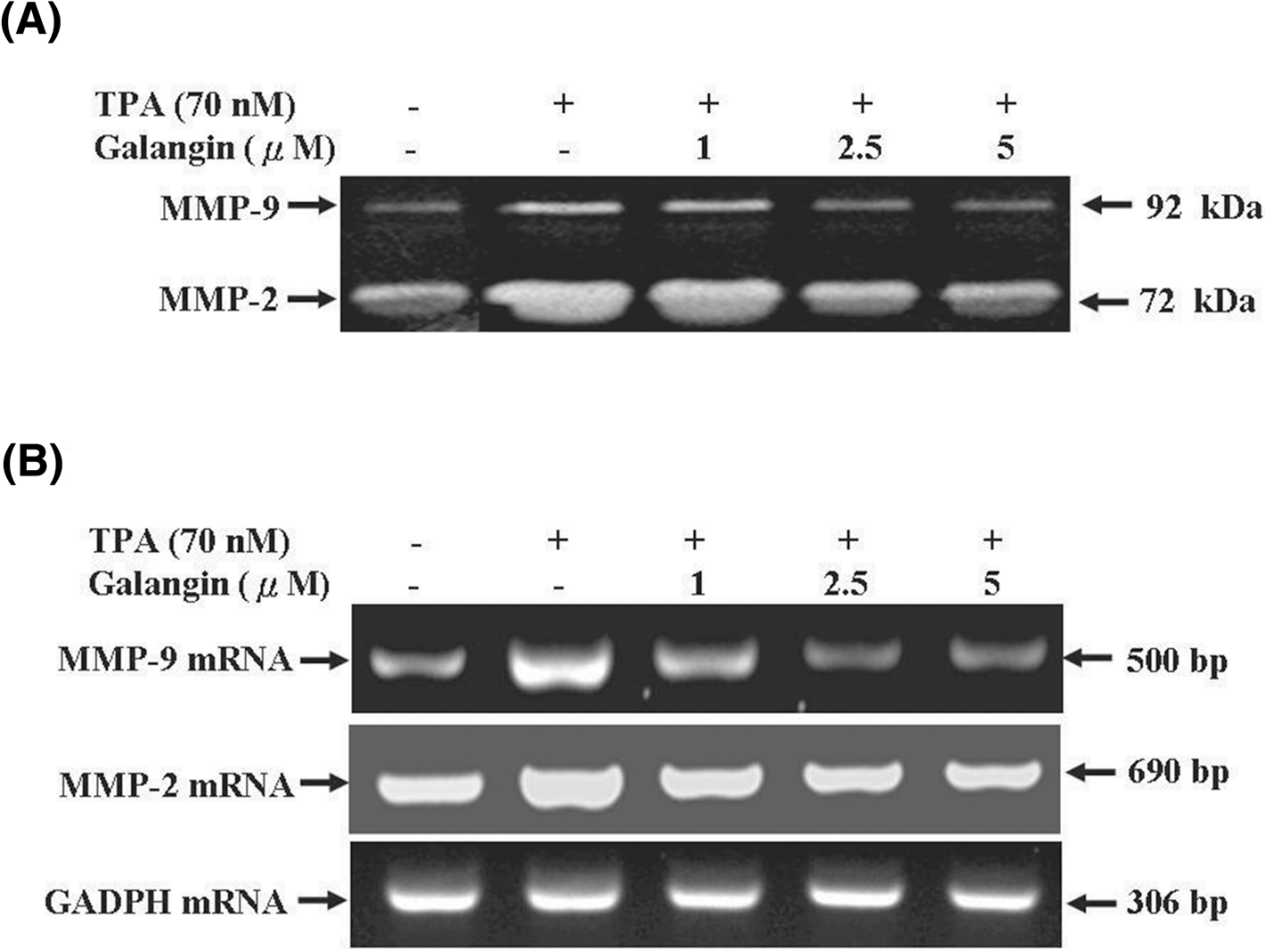
**Effect of galangin on TPA-induced activity and expression of MMP-2/MMP-9 in HepG2 cells. (A)** Cells were treated with 70 nM TPA for 12 h in serum-free medium and then incubated in various concentrations of galangin (0, 1, 2.5, and 5 μM) for 24 h. The conditioned media were collected and MMP-2/MMP-9 activities were determined by gelatin zymography. **(B)** Cells were treated with 70 nM TPA for 12 h and incubated in various concentrations of galangin (0, 1, 2.5, and 5 μM) for 12 h. And then, the RNA samples were extracted and subjected to a semi-quantitative RT-PCR for MMP-2 and MMP-9 with GADPH being an internal control.

ECM degradation is crucial to cellular invasion, suggesting that matrix-degrading proteinases determine whether MMP-2 and MMP-9 are involved in the inhibition of invasion, motility, and adhesion of cancer cells by galangin; therefore, we investigated the inhibitory effect of galangin on TPA-induced MMP-2/-9 enzyme activity by using gelatin zymography under a condition of serum starvation. Figure 3A showed that TPA significantly increased MMP-2 and MMP-9 activity. In addition, galangin inhibited MMP-2 and MMP-9 activity that was stimulated by TPA in a concentration-dependent manner. To determine whether the inhibition of MMP-2 and MMP-9 enzyme activity by galangin was caused by the reduction of the transcription level, we performed a RT-PCR and observed the mRNA expression of MMP-2 and MMP-9. As shown in Figure 3B, galangin reduced the TPA-induced MMP-2 and MMP-9 mRNA expression of HepG2 cells in a dose-dependent manner. The data indicated that galangin prevents the transcription of MMP-2 and MMP-9 in response to TPA. These results suggested that the antimetastatic effect of galangin was related to the inhibition of enzymatically degradative processes of tumour metastasis.

### Galangin inhibited TPA-induced the protein levels of PKCα, PKCδ, and p-ERK1/2 in HepG2 cells

**Figure 4:**
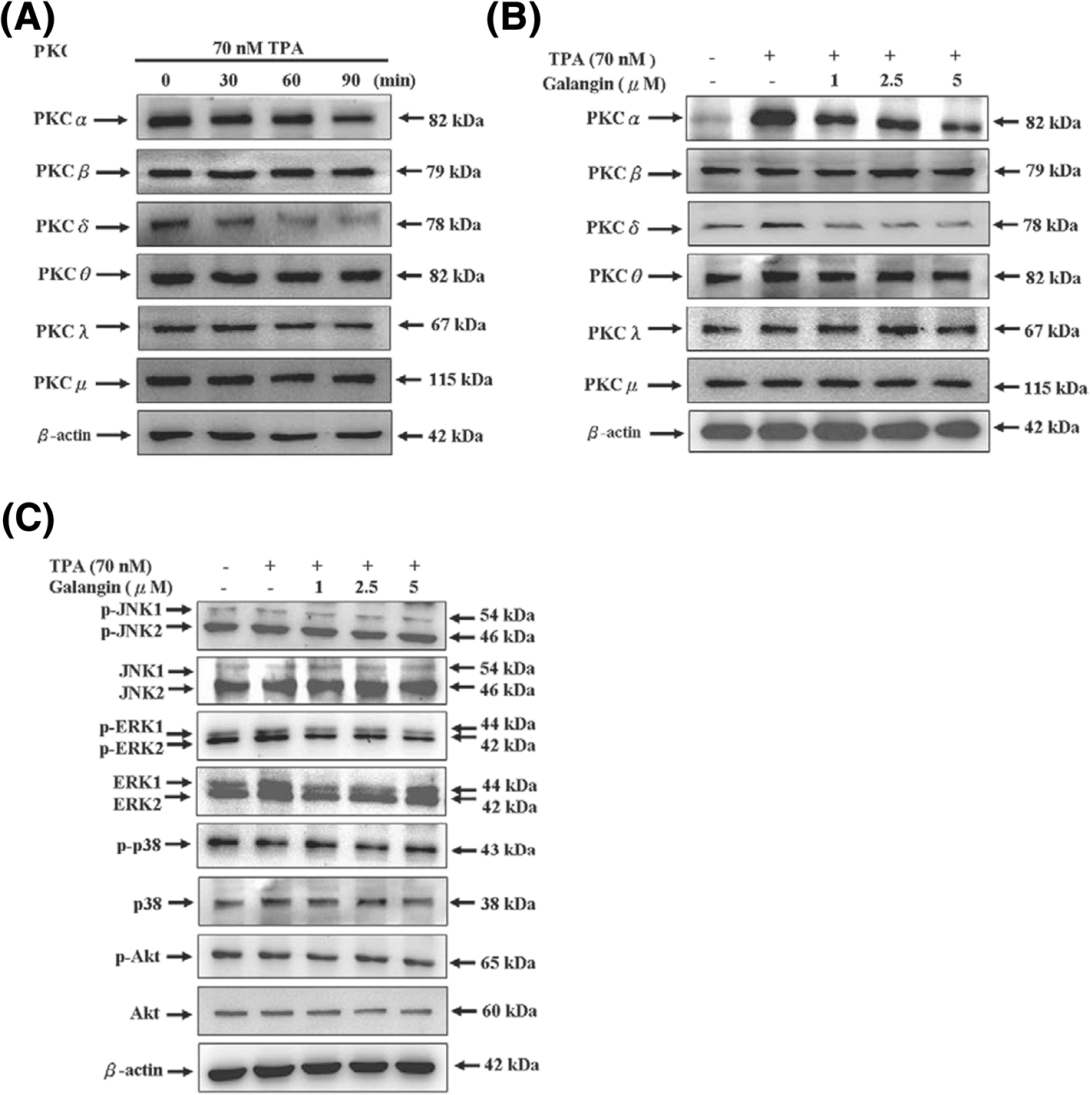
**Effect of galangin on TPA-induced the PKCα** **, PKCδ** **activation and ERK phosphorylation in HepG2 cells. (A)** Cells were treated with TPA (70 nM) for various times, the protein levels of PKCα, PKCβ, PKCδ, PKCθ, PKCλ, and PKCμ in the membrane fraction were analyzed. **(B)** Cells were treated with various concentrations (0, 1, 2.5, and 5 μM) of galangin in the presence or absence of TPA (70 nM) for 6 h, the protein levels of PKCα, PKCβ, PKCδ, PKCθ, PKCλ, and PKCμ in the membrane fraction was analysed. **(C)** Cells were treated with TPA (70 nM) for 2 h in various concentrations (0, 1, 2.5, and 5 μM) of galangin for 6 h. The JNK phosphorylation, JNK, ERK phosphorylation, ERK, p38 phosphorylation, p38, Akt phosphorylation, and Akt were analysed by Western blotting. β-Actin was used as a loading control. The relative density of phosphorylated forms of JNK, ERK, and p38 were normalized to total values of JNK, ERK, and p38, which were determined by densitometric analysis.

We investigated whether galangin inhibited TPA-induced the expression of PKC family members in HepG2 cells. This result of Western blot revealed that galangin inhibited TPA-induced the protein levels of PKCα and PKCδ in the time- and dose-dependent manners. No substantial changes were observed in the protein levels of PKCβ, PKCθ, PKCλ, and PKCμ. (Figure 4A and B).

Furthermore, we investigated the effect of galangin on the phosphorylation of JNK1/2, ERK1/2, p38, and Akt in cells stimulated by 70 nM TPA for 2 h. HepG2 cells were then treated with various concentrations of galangin for 6 h. Figure 4C showed that galangin significantly inhibited TPA-induced ERK1/2 activation, indicated by the decrease in the phosphorylation of ERK1/2. However, galangin did not significantly affect phospho-JNK, phospho-p38, or phospho-Akt activity. Moreover, the total protein levels of JNK1/2, ERK1/2, p38, and Akt did not change after TPA and/or galangin treatment.

### Galangin-inhibited TPA-induced NF-κB and AP-1 transcriptional activation in HepG2 cells

**Figure 5:**
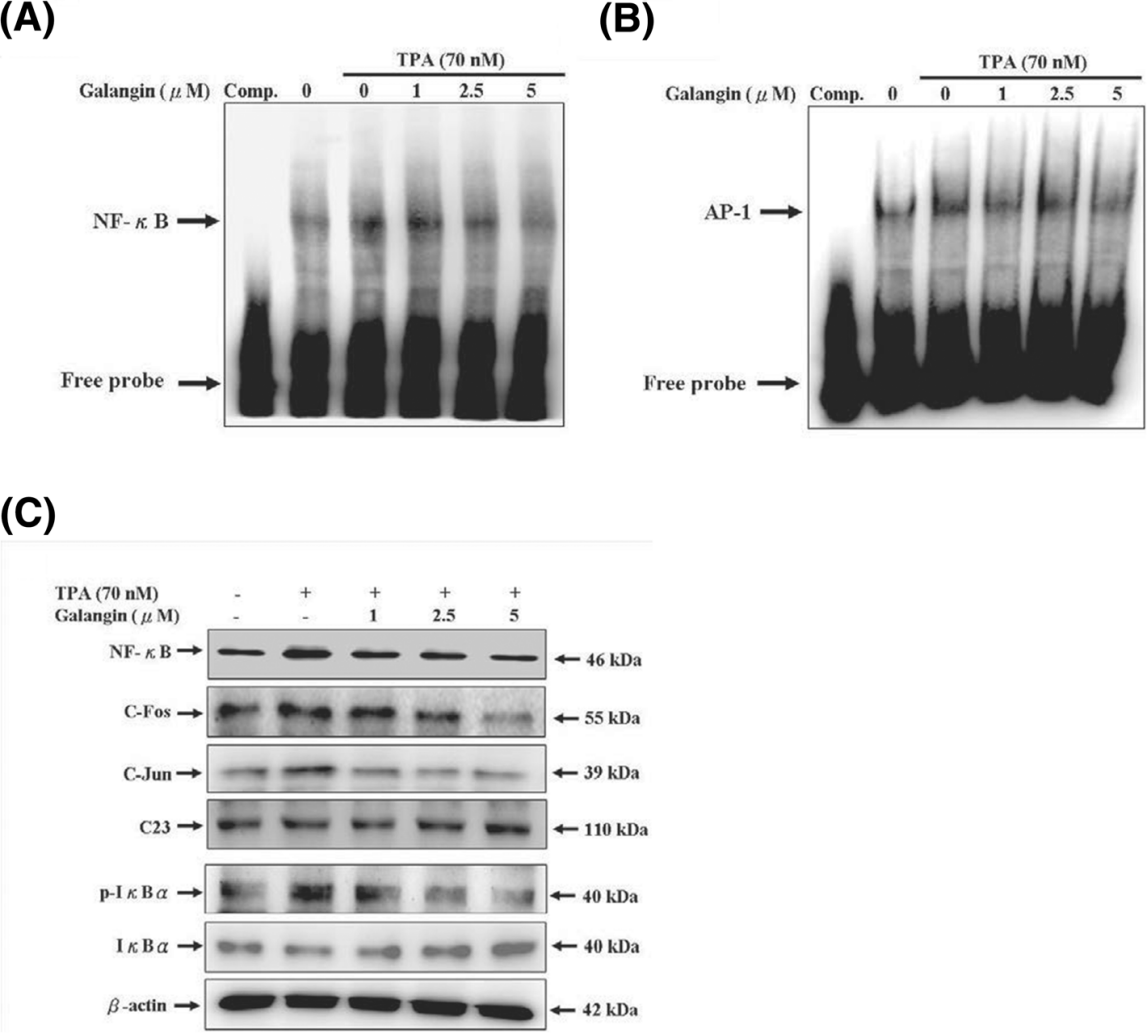
**Effect of galangin on the TPA-induced the DNA-binding activity of NF-κ** **B and AP-1/expressions of NF-κ** **B, c-Fos, c-Jun/Iκ** **Bα** **phosphorylation and degradation in HepG2 cells.** Nuclear extracts were prepared from HepG2 cells that treated with various concentrations of galangin (0, 1, 2.5, and 5 μM) in the presence of TPA (70 nM) for 12 h, and then used to analyze **(A)** NF-κB and **(B)** AP-1 DNA-binding activity by EMSA, as described in “Materials and methods” section. Lane 1: nuclear extracts incubated with 100-fold excess unlabeled consensus oligonucleotide (comp.) to confirm the binding specificity. Lane 2 represents nuclear extract from HepG2 cells in the absence of TPA (negative control). **(C)** Nuclear or cytosolic extracts were subjected to SDS-PAGE followed by western blotting with specific antobodies (anti-NF-κB, anti-c-Fos, anti-c-Jun anti-p-IκBα, anti-IκBα). C23 and β-actin were used as internal control.

NF-κB and AP-1 are the primary transcription factors that regulate the expression of MMP enzymes in cells. To investigate the association between NF-κB and AP-1 and the anticarcinogenic property of galangin, we analysed the ability of NF-κB and AP-1 bind to MMP-2/9 promoters by using an EMSA. As shown in Figure 5A and B, the NF-κB and AP-1 DNA binding activity was dramatically increased by TPA (70 nM) treatment, and the TPA-stimulated NF-κB and AP-1 DNA binding activity was strongly inhibited by galangin at 5 μM concentration. Furthermore, the expressions of NF-κB, c-Fos, and c-Jun in nuclear extracts was analysed using Western blotting to assess the possible inhibitory effect of galangin. Figure 5C shows that the nuclear levels of NF-κB, c-Fos, and c-Jun were dramatically diminished by TPA (70 nM) treatment when galangin doses of 1, 2.5, and 5 μM were applied, and the TPA-stimulated nuclear protein expressions NF-κB, c-Fos, and c-Jun were strongly inhibited by galangin at 5 μM concentration. In addition, NF-κB was activated through the phosphorylation of IκBα releasing NF-κB for nuclear translocation, and for binding to the promoter sites of target genes. The results showed that galangin blocked TPA-induced IκBα degradation by inhibiting the phosphorylation of IκBα. Moreover, the intensity of the bands on the Western blotting reflected that galangin at a concentration of > 1 μM enhanced IκBα protein expression.

## Discussion

For the past several decades, despite evolving management and novel therapies for various types of cancers, the prognosis for several types of cancers remain poor. Because increasingly more researchers have dedicated themselves to tumour research, the mechanisms underlying the oncogenesis and metastasis of cancer have gradually been revealed, and additional anticarcinogenic compounds have been discovered. Consequently, cancer chemoprevention for inhibiting the progression of cancer by using certain compounds or mixtures, has become a potentially viable method for treating cancer. Galangin is type of flavonoid with the highest concentration levels amongst *Alpinia officinarum*. Flavonoids are a vast group of heterogeneous polyphenols that are thought to exert positive effects, including cancer prevention, on human health. Various studies have shown that glangin exerts pleiotropic anticancer effects, including preventive, anticarcinogenic, and antiproliferative effects. In particular, glangin influences several processes and plays a crucial roles in regulating various molecular targets, including NF-κB [25,26], Smads [27], peroxisome proliferator-activated receptor γ (PPARγ) [28] transcription factors, tumour necrosis factor-alpha (TNF-α), interleukins, intercellular adhesion molecule-1 (ICAM-1), cyclooxygenase-2 (COX-2) [29-32], JNK, p38 [25], and ERK [26]. Metastasis is a critical characteristic of malignant tumours that requires a series of signal transductions. When these signal pathways are blocked with drugs or natural compounds, the spread of cancer may be inhibited. The purpose of this study was to determine whether galangin can inhibit HepG2 cancer cell invasion, migration and adhesion, and to elucidate its mechanism at a molecular level.

PKCs were shown to play key roles in various cellular responses, including the regulation of gene expression and the effects exerted on the cytoskeleton, cell growth, and differentiation [33]. At least PKCs have recognised and are typically distributed into three classes, namely conventional (PKCα, βI, βII, γ), novel (δ, ε, ε’, η, θ, μ), and atypical (ζ, λ/ι) PKCs. Amongst these three classes, the activity of conventional PKCs depends on Ca^+2^, diacyglycerol (DAG), or a phorbol ester analog of DNA such as TPA [34-37]. Isozymes of the PKC family exert various actions in cellular proliferation through a complex network of signal transduction. Previous studied have indicated that PKCα is a crucial signalling molecule for MMP expression in tumor cells [38,39]. In addition, PKCδ was shown to activate MMP expression [40]. We found that galangin inhibited the mRNA expressions and enzymic activity of MMP-2 and MMP-9 in HepG2 cells. These results suggested that decreases in the protein expression of PKCα and PKCδ are vital to the suppression of MMP-2 and MMP-9 expression by galangin in HepG2cells. The activation of PKC can lead to the activation of MAPKs and the PI3K/Akt pathway. Our results revealed that long-term exposure to 70 nM TPA leads to downregulation of PKC-α and PKC-δ in HepG2 cells, indicating that TPA is involved in the ERK1/2 signal transduction pathway. These findings indicated that galangin inhibited TPA-induced MMP-2 and MMP-9 expression through the suppression of the PKC/ERK pathway in HepG2 cells.

The transcription of MMP-2/-9 genes is regulated by upstream sequences, including motifs corresponding to NF-κB and AP-1 binding sites. NF-κB and AP-1 are activated in numerous pathological processes, including inflammation, cancer-cell adhesion, invasion, metastasis, and angiogenesis. Previous studies have reported that the MMP-2/-9 system plays a crucial role in breast cancer growth, invasion, and metastasis. In this study, we found that treating HepG2 cells with galangin resulted in inhibition of NF-κB and AP-1 DNA binding activity, which was accompanied by inhibition of the nuclear translocation of these factors.

## Conclusions

In conclusion, our results reveal the first scientific evidence showing that the suppression of the PKC/ERK pathway by galangin downregulates TPA-induced MMP-2/-9 activation, thereby inhibiting migration and invasion in human HepG2 cells. Thus, galangin may be an effective ingredient in agents developed for preventing cancer metastasis.
